# Nonpuerperal uterine inversion due to adenomyosis: A case report and a literature review

**DOI:** 10.1002/ccr3.2530

**Published:** 2019-11-05

**Authors:** Philippe Merviel, Sandrine Guilbert, Coline Abou Hassan, Isabelle Thomas‐Kergastel, Virginie Conan‐Charlet, Pascale Marcorelles, Pierre‐François Dupré

**Affiliations:** ^1^ Gynecology and Obstetrics Department Brest University Hospital Brest France; ^2^ Radiology Department Brest University Hospital Brest France; ^3^ Histopathology Department Brest University Hospital Brest France

**Keywords:** adenomyosis, inversion, MRI, uterus

## Abstract

Nonpuerperal uterine inversion is a very rare event. We reported on the first ever case of nonpuerperal uterine inversion due to adenomyosis. Magnetic resonance imaging is recommended in cases with an unusual vaginal mass, so that this possible uterine etiology can be taken into consideration.

## Graphical abstract

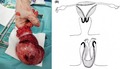

## 1 INTRODUCTION

Although puerperal uterine inversion is rare (1 case per ~30 000 deliveries),^1^ nonpuerperal uterine inversion is even rarer: Only 150 such cases (accounting for 17% of all inversions) were described.^2^ Nonpuerperal uterine inversions are due to myoma or polyps or uterine tumors (leiomyosarcoma, rhabdomyosarcoma, mixed Müllerian tumor, cervical or endometrial carcinoma, carcinoma).^2^ Here, we report on the first case of nonpuerperal uterine inversion caused by adenomyosis.

## 2 CLINICAL CASE

**Figure 1:**
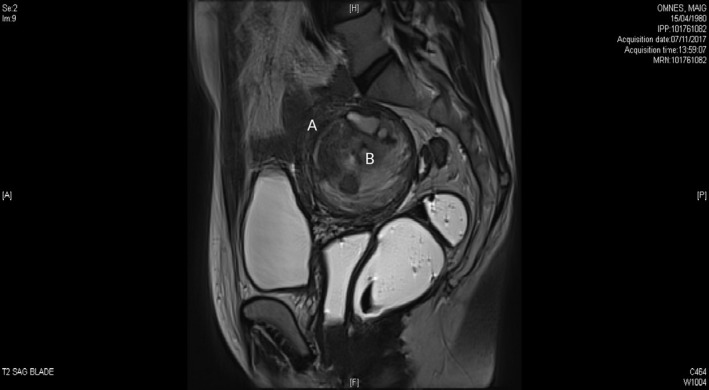
Sagittal cut in MRI—T2‐weighted sequence: The uterine mass (B) is heterogeneous, distorting the uterine cavity visible in the form of a thin‐edged in antero‐superior (A)

**Figure 2:**
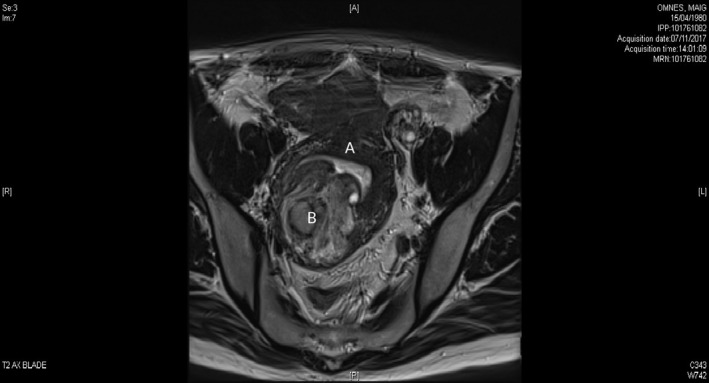
Cross section in MRI—T2‐weighted sequence: The uterine mass (B) deforms the uterine cavity, visible in the form of a parasol figure (A)

A 38‐year‐old primiparous woman with an unremarkable medical history consulted in September 2017 for pelvic pain, menorrhagia, dysmenorrhea, and dyspareunia—suggesting the presence of adenomyosis. Magnetic resonance imaging showed the uterus with a longest dimension of 8.6 cm and an anteroposterior diameter of 7.3 cm. The endometrium had a normal aspect and thickness. A nodular lesion (measuring 6.5‐5.3 cm) was present in the posterior myometrial wall. The lesion was heterogeneous (with areas of hyperintensity in a T2‐weighted MRI sequence) and contained a large number of cyst‐like structures. In a T1‐weighted sequence, some of the cysts had a hyperintensity pattern consist with hemorrhage, whereas others appeared to be filled with fluid. Thickening of the junctional zone was suggestive of adenomyotic damage. In contrast‐enhanced sequences, the lesion appeared to be well delimited and had intense but not suspicious foci of uptake. On this basis, we diagnosed interstitial myoma with focal hemorrhagic cysts (Figures 1 and 2). The patient attended a follow‐up consultation in December 2017. A cervical smear was normal, and a three‐month course of ulipristal acetate 5 mg/d (Esmya^®^, Gedeon Richter) was initiated. Due to poor tolerance (nausea and vomiting) and lack of effectiveness on bleeding, the patient stopped taking the medication. In August 2018, she consulted for a second opinion. A clinical examination showed a retroverted uterus but no vaginal prolapse. Various treatment options (myomectomy, adenomyomectomy, and hysterectomy) were discussed with the patient but none was immediately chosen. In January 2019, the woman (who worked in the catering industry) returned to our clinic complaining of abnormal bleeding for 3 weeks a month and pelvic heaviness. A pelvic examination revealed a large intravaginal mass; it measured between 6 and 7 cm, protruded from the cervix, and had the appearance of myoma. Given the recurrence of abnormal bleeding, the pain, and the aspect of the vagina, the woman agreed to have a hysterectomy without adnexectomy. Three days after this consultation, the woman was admitted to the emergency department for acute pelvic pain (rated at 9 out of 10 on a visual analogue scale) and bleeding. The very intense pain suggested torsion or necrobiosis of the myoma. The woman's pain was relieved in part by treatment with paracetamol, a nonsteroidal anti‐inflammatory, and a muscle relaxant. This treatment was combined with hypnosis sessions, which increased the patient's pain tolerance. The pain level fell over the following 48 hours, and surgery was performed three days after the patient had been admitted to the emergency department.

**Figure 3:**
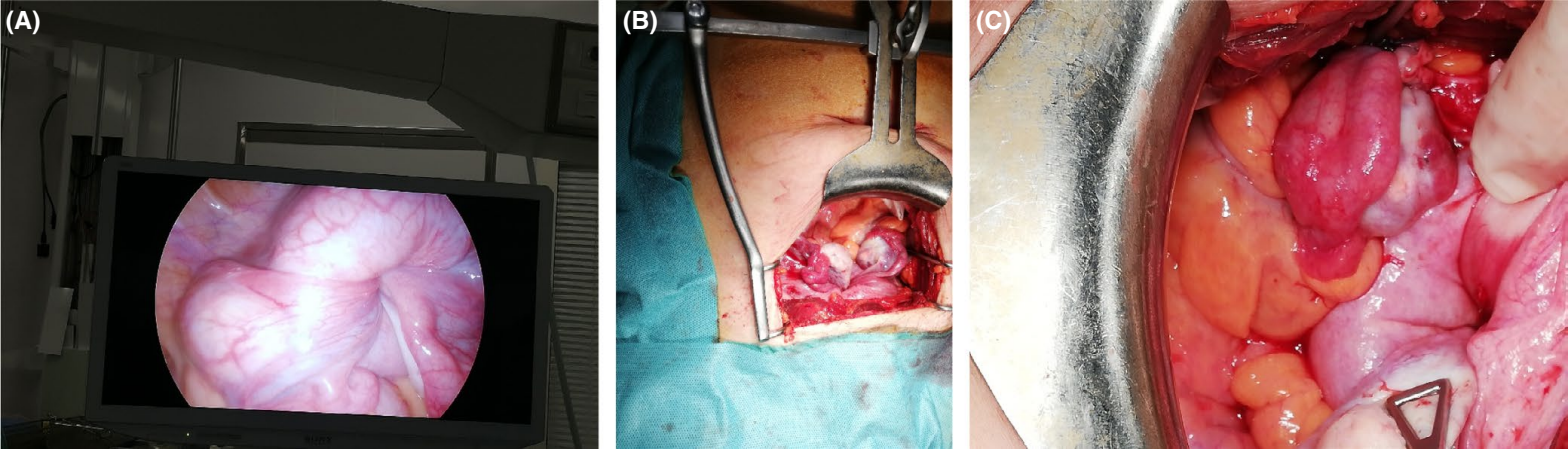
Per‐opérative views (A, by laparoscopy; B, C, in the process of laparotomy). A, both adnexal structures and bladder sound invaginated to the uterine fundus. The uterus is not visible. B, surgical appearance at the beginning of laparotomy. C, The left adnexal structure has been ligatured and then severed, and the right ovary is spotted by a triangular clamp. The fundic invagination of the uterus and bladder begins to be more visible

**Figure 4:**
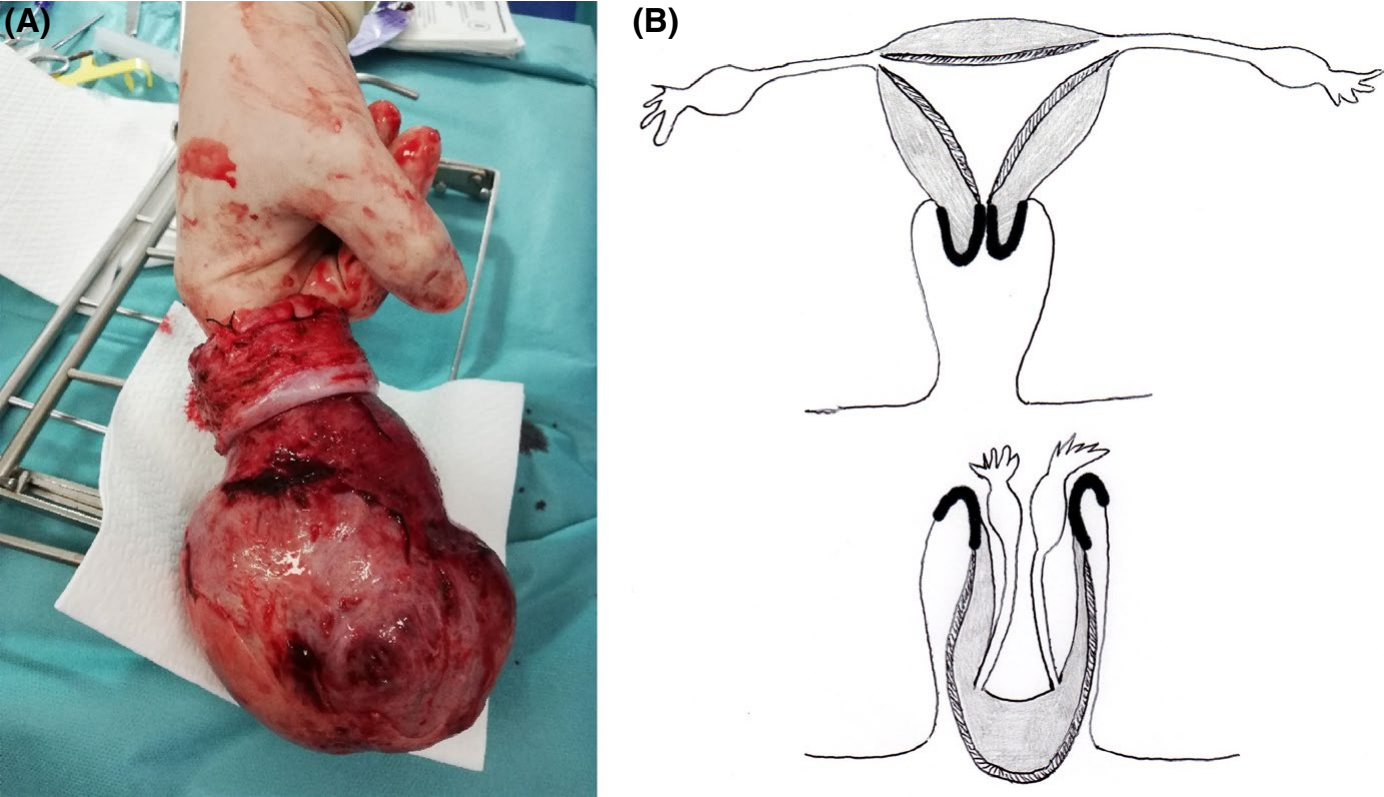
Operative specimen (uterine body and cervix): The operator's finger is located in the fundic invagination of the uterus. The outer part of the uterine body is covered with endometrium, and the cervix is recognizable by its whitish appearance. The diagram shows the different uterine structures, before and after the inversion

During the (initially laparoscopic) surgical procedure, we observed an invagination of the Fallopian tubes and the ovarian and round ligaments. It was impossible to see the uterus (Figure 3A). We then decided to perform a laparotomy, which revealed total uterine inversion (Figure 3B). The round and ovarian ligaments and the Fallopian tubes (which were ligated and then severed (Figure 3C)) were released in a stepwise procedure. An intraoperative methylene blue test showed that the upper pole of the bladder was completely inverted. By opening the peritoneum of the bladder, we lifted up the uterus and thus released the uterine arteries. Hysterectomy was then performed. Figure 4 shows the inverted uterus after extraction: the operator's finger (measuring about 9 cm in length) is located within the invaginated fundus. The postoperative course was uneventful, and the patient was discharged on postoperative day 3.

**Figure 5:**
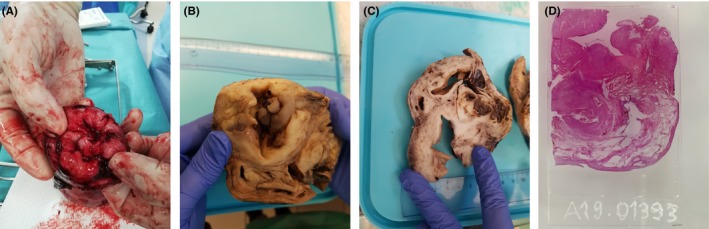
Per‐opérative and histopathological aspects (macroscopic and microscopic) of the uterine body. A, per‐opérative aspect of the uterine fundus: the invagination. The slices of proximal sections of the round and utero‐ovarian ligaments and fallopian tubes is distinguished in the center of invagination. B, same location as 5A but in macroscopic histopathological examination. C, macroscopic histopathological aspect of the uterine body: The invagination is distinguished (between the two fingers, covered with the peritoneum) and the outer part of the specimen, covered with endometrium. Several adenoma cysts are visualized, three of which are filled with brownish blood, and one is unfilled and sagging (diameter of 35 mm). D, same localization as 5C but in microscopic histopathological examination. AC, adenomyosis cyst; E, endometrium; P, peritoneum

Histopathological assessments of the cervix (Figure 5A: a per‐opérative view; 5B: a macroscopic view) and the uterine corpus (Figure 5C: a macroscopic view; 5D: a microscopic view) were performed. The resected uterus was 12.5 cm long, 8 cm wide, and 7 cm thick. The uterus was bounded by the endometrial mucosa, and the peritoneum was located in the center of the invaginated area. The very extensive adenomyotic lesions contained a number of large cysts measuring up to 35 mm in diameter. There were no signs of malignancy. On the basis of this assessment, the final diagnosis was uterine inversion caused by major adenomyotic damage.

## 3 DISCUSSION

Most cases of nonpuerperal uterine inversion occur in women over the age of 40; in Gomez‐Lobo et al's study, only 4 cases appeared before that age.^2^ We analyzed 40 cases of nonpuerperal uterine inversion reported in the literature between 1897 and 2018.^3^, ^4^, ^5^ The woman's age ranged from 15 to 88 (mean ± standard deviation: 41.5 ± 17); 13 were primiparous, 25 were multiparous, and 2 had never had sexual intercourse. The lesion size ranged from 2.9 cm (the teratoma) to 18 cm (mean: 8.5 ± 4.6 cm). The uterine inversion was caused by malignancy in 26 cases (65%), including sarcoma (leiomyosarcoma, rhabdomyosarcoma, and fibrosarcoma [n = 20]), mixed Müllerian tumors (n = 5), and cervical adenocarcinoma (n = 1). Thirteen cases featured one or more myomas, and there was one case of teratoma of the uterus (in a 15‐year‐old). The women's mean age was 44.3 for sarcoma, 46.9 for myoma, and 30.5 for the other lesions. Our patient was 38 years old at the time of diagnosis, primiparous, and a histopathological assessment did not reveal any signs of malignancy. The treatment consisted of hysterectomy (n = 31, 77.5%; 26 with abdominal access, and 5 with vaginal access), conservative surgery (for teratoma and myoma; n = 3), and biopsy followed by radiotherapy (for sarcoma, n = 2; publications before 1950). Our review confirmed that the present report is the first to have described nonpuerperal uterine inversion due to adenomyosis.

The inversion is said to be incomplete when the uterine fundus does not pass through the cervix, complete when the uterine corpus passes through the cervix, and total when both the uterus and vagina are inverted. The most common clinical signs of uterine inversion are anemia associated with hemorrhage, abdominal pain, the presence of a vaginal mass, and (in some cases) urethral obstruction. In a clinical examination with bimanual palpation, the uterine fundus is not palpable. Although our patient had experienced menorrhagia and pelvic pain for more than a year, the intravaginal mass (inversion) and the onset of acute pain (corresponding to aggravation of the inversion and, probably, to ischemia of the uterus) occurred only a few days before surgery. On the basis of the MRI findings, we initially thought that the intravaginal mass was perhaps a prolapsed myoma. The two other differential diagnoses (cervical cancer and uterine prolapse) were, respectively, ruled out by the normal cervical smear test 9 months previously and our inability to see the cervix.

The pathophysiology of uterine inversion is thought to be related to the coincidence of several factors: a thin uterine wall, a large, rapidly growing, pedunculated tumor in the fundus, and gradual dilatation of the cervix after that of the uterine cavity.^6^, ^7^ The mass's weight and the occurrence of uterine contractions also have a role, and abnormally high intra‐abdominal pressure (due to coughing, sneezing, constipation, etc) may promote uterine inversion.^8^ In the present case, the lesion was nonpedicled, located behind the fundus, and (if one compares its size on MRI with that determined during the histopathological examination) slow‐growing. The cervix was normal (according to a smear test in March 2018 and an examination in August 2018), and the uterus was not fully inverted. If one considers that presence of blood in the adenomyotic cysts led to inversion, the estimated extra weight of the uterine fundus was around 25 grams (4 π/3 × radius^3^ × the density of blood [1.066]). Our patient did not report any episodes of coughing but had vomited while taking ulipristal acetate (between November 2017 and March 2018), which would have generated abnormally high intra‐abdominal pressure. However, palpation of the uterus was normal at the clinical examinations in March and August 2018.

We did not perform medical imaging (ultrasound or MRI) during the episode of acute abdominal pain that occurred a few days before surgery. Myoma was diagnosed in November 2017 on the basis of the MRI findings and the clinical aspect of the intravaginal mass, which was strongly suggestive of a myoma that had prolapsed through the cervix. We should probably have performed MRI again in January 2018; this might have revealed the signs described by Moulding,^9^ that is the presence of round ligaments and Fallopian tubes over the central part of the uterine fundus, and their invagination into it. Provided that a histological analysis confirms the benign nature of the lesion, conservative treatment may be considered when the inversion is incomplete or reducible or when it occurs in a young woman. In other cases, the usual treatment is hysterectomy. Before the episode of acute pain, our patient had agreed to have a hysterectomy for myoma and adenomyosis, rather than drug therapy for resolution of the bleeding. The hysterectomy was carried out cautiously, in order to avoid any organ damage; in particular, we ensured that the ureters were visible throughout the operation.^10^

## 4 CONCLUSIONS

We reported on the first ever case of nonpuerperal uterine inversion due to adenomyosis. Magnetic resonance imaging might have enabled earlier diagnosis of this complication—especially in view of the acute pelvic pain experienced by the patient. The treatment would have been the same, however, given the irreducible nature of the inversion, the presence of hemorrhagic adenomyosis, and the absence of plans for further pregnancies.

## CONFLICT OF INTEREST

None declared.

## AUTHOR CONTRIBUTIONS

Philippe Merviel: served as the gynecologist and the surgeon in this case, and Head of the Gynecology and Obstetrics department of the Brest University Hospital; conceived the manuscript; and revised the article. Sandrine Guilbert: served as the gynecologist at the start of the case history. Coline Abou Hassan: served as the medical student and assistant during the surgery in this case. Isabelle Thomas‐Kergastel: served as the radiologist (MRI) in this case. Virginie Conan‐Charlet: served as the pathologist in this case. Pascale Marcorelles: served as the pathologist referent and Head of the Histopathology department of the Brest University Hospital. Pierre‐François Dupré: served as the gynecologist and contributed to conceive the manuscript.
